# Supplementary material for: Cannabinoid inhibition of mechanosensitive K+ channels
Source: bioRxiv. 2024 Dec 10:2024.12.09.627564. Preprint. [Version 1] doi: 10.1101/2024.12.09.627564 (PMC11661169; doi:10.1101/2024.12.09.627564)
Supplement: Supplement 1 [file NIHPP2024.12.09.627564v1-supplement-1.pdf]

bioRxiv preprint doi: https://doi.org/10.1101/2024.12.09.627564; this version posted December 10, 2024. The copyright holder for this preprint (which was not certified by peer review) is the author/funder, who has granted bioRxiv a license to display the preprint in perpetuity. It is made available under aCC-BY 4.0 International license.

Supplementary Figure 1 – Comparison of CBD inhibition of disease-causing TRAAK variants.

(a) Overlaid dose-response curves and **(b)** comparison of fit IC_{50} values (wild-type: 145±4 nM, P259L: 156±8 nM, G165E: 162±8 nM, G165R: 356±18 nM, A198E: 496±26 nM, A270P: 1.12±0.04 µM, and G158D: 1.03 ± 0.22 µM (mean ± sem, n = 9, 4, 4, 6, 7, 8 and 5 patches, respectively). Differences assessed with Brown-Forsythe and Welch Annova with Dunnet correction for multiple comparisons, ***P<0.001, ****p<0.0001, n.s. not significant.

Supplementary Figure 2 – CBD inhibition reduces mechanical activation of TRAAK.

(a-e) Example current traces from a TRAAK-containing excised patch in response to negative pressure at varying concentrations of CBD ranging from **(a)** 0µM, **(b)** 500nM, **(c)** 1µM, **(d)** 10µM, and **(e)** following washout. **(f)** Peak current versus pressure from data in **(a-e)**.

bioRxiv preprint doi: https://doi.org/10.1101/2024.12.09.627564; this version posted December 10, 2024. The copyright holder for this preprint (which was not certified by peer review) is the author/funder, who has granted bioRxiv a license to display the preprint in perpetuity. It is made available under aCC-BY 4.0 International license.

Supplementary Figure 3 – CBD treatment does not alter membrane tension.

(a) Representative images of membrane patches under -10 (left) and -50 mmHg (right) pressure in 0 μM CBD (top) and 50μM CBD (bottom). **(b)** Calculated membrane tension versus applied negative pressure in 0 μM CBD (light green) and 50 μM CBD (dark green). Asterisks indicate data from images shown in **(a). (c)** Comparison of tension generated at matching pressure steps. Data from 10 paired recordings from three different patches are shown ($p = 0.58$, two-tailed paired t-test, not significant).

Supplementary Figure 4 – CBD inhibition of *Danio rerio* TREK-1.

(a) Dose-response curve. Data from individual patches are plotted with different shapes. Solid line is the fit to a four parameter logistic curve with 95% confidence interval shaded. IC_{50} = 11.1±1.3μM (mean±sem, n = 4 patches). **(b)** Representative current-voltage relationship from a *Danio rerio* TREK-1-containing patch before and after application of 30 μM CBD.

Supplementary Figure 5 – Cryo-EM data processing pipeline for TREK-1:CBD.

(a,b) Representative micrograph and initial cryo-EM data processing from each dataset, **(c)** cryo-EM data processing pipeline from merged particle stack, and **(d)** representative 2D class averages and reconstructed map from final particle stack.

Supplementary Figure 6 – Cryo-EM structure validation for TREK-1:CBD

Final sharpened map colored by local resolution and viewed from **(a)** the membrane plane and **(b)** the cytoplasmic side. **(c)** View angle distribution of final particle stack. **(d)** Fourier shell correlation (FSC) versus resolution between half maps from final refinement.

Supplementary Figure 7 – CBD-bound TREK-1 is stabilized in the TM4-down conformation.

(a) Final map and structure of TREK-1:CBD focused on TM4. **(b)** Final map and structure of apo TREK-1 focused on TM4. **(c)** Final map and structure of TREK-1:POPE focused on TM4. **(d)** comparison of TM4 conformation in apo TREK-1 (purple) and TREK-1:CBD (green) and **(e)** TREK-1:POPE.

Supplementary Figure 8 – CBD-induced changes in the TREK-1 selectivity filter.

bioRxiv preprint doi: https://doi.org/10.1101/2024.12.09.627564; this version posted December 10, 2024. The copyright holder for this preprint (which was not certified by peer review) is the author/funder, who has granted bioRxiv a license to display the preprint in perpetuity. It is made available under aCC-BY 4.0 International license.

Comparison of the TREK-1 selectivity filter and K^+ density from **(a)** TREK-1:CBD, **(b)** apo TREK:1, and **(c)** TREK-1:POPE structures. **(d)** Overlaid selectivity filters from apo TREK-1 (purple) and TREK-1:CBD (green) structures.

bioRxiv preprint doi: https://doi.org/10.1101/2024.12.09.627564; this version posted December 10, 2024. The copyright holder for this preprint (which was not certified by peer review) is the author/funder, who has granted bioRxiv a license to display the preprint in perpetuity. It is made available under aCC-BY 4.0 International license.

Supplementary Table 1 - Cryo-EM data collection, refinement, and validation statistics

	*dr*TREK-1:CBD	
PDB ID	9DBR	
EMDB ID	46725	
EMPIAR ID		
**Data collection**	**Collection 1**	**Collection 2**
Total movies	9,046	8,470
Magnification	105,000x	
Voltage (kV)	300	
Electron exposure ($e^-/Å^2$)	50	
Defocus range (um)	-0.5 to -1.8	
Pixel size ($Å^2$)	0.848	
**Processing**		
Initial particle images (no.)	261,227	228,680
Joint Particle Count	484,500	
Final particle images (no.)	117,609	
Map resolution Masked (Å, FSC = 0.143)	3.94	
Symmetry imposed	C1	
**Refinement**		
Model resolution (Å, FSC = 0.143)	3.9	
Map-sharpening B factor ($Å^2$)	190.5	
Composition		
Number of atoms	4037	
Number of protein residues	510	
Number of ligands	3	
RMS deviations		
Bond lengths (Å)	0.004	

bioRxiv preprint doi: https://doi.org/10.1101/2024.12.09.627564; this version posted December 10, 2024. The copyright holder for this preprint (which was not certified by peer review) is the author/funder, who has granted bioRxiv a license to display the preprint in perpetuity. It is made available under aCC-BY 4.0 International license.

Bond angles (Å)	0.567
Validation	
MolProbity score	1.44
Clashscore	5.14
Ramachandran plot	
Favored (%)	97.04
Allowed (%)	2.96
Disallowed (%)	0
Rotamer outliers (%)	0
Mean B factor (Å^2)	
Protein	135.78
Ligand	93.61

bioRxiv preprint doi: https://doi.org/10.1101/2024.12.09.627564; this version posted December 10, 2024. The copyright holder for this preprint (which was not certified by peer review) is the author/funder, who has granted bioRxiv a license to display the preprint in perpetuity. It is made available under aCC-BY 4.0 International license.

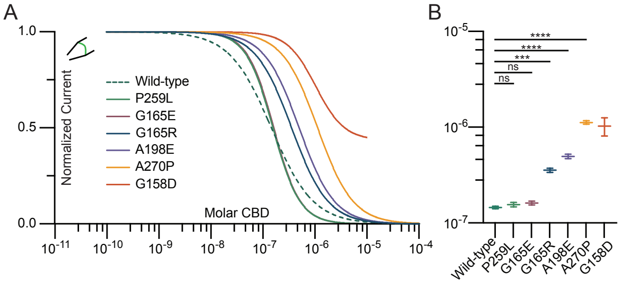

Supplementary Figure 1 – Comparison of CBD inhibition of disease-causing TRAAK variants.
(a) Overlaid dose-response curves and (b) comparison of fit IC_{50} values (wild-type: 145±4 nM, P259L: 156±8 nM, G165E: 162±8 nM, G165R: 356±18 nM, A198E: 496±26 nM, A270P: 1.12±0.04 μM, and G158D: 1.03 ± 0.22 μM (mean ± sem, n = 9, 4, 4, 6, 7, 8 and 5 patches, respectively). Differences assessed with Brown-Forsythe and Welch Annova with Dunnet correction for multiple comparisons, ***P<0.001, ****p<0.0001, n.s. not significant.

bioRxiv preprint doi: https://doi.org/10.1101/2024.12.09.627564; this version posted December 10, 2024. The copyright holder for this preprint (which was not certified by peer review) is the author/funder, who has granted bioRxiv a license to display the preprint in perpetuity. It is made available under aCC-BY 4.0 International license.

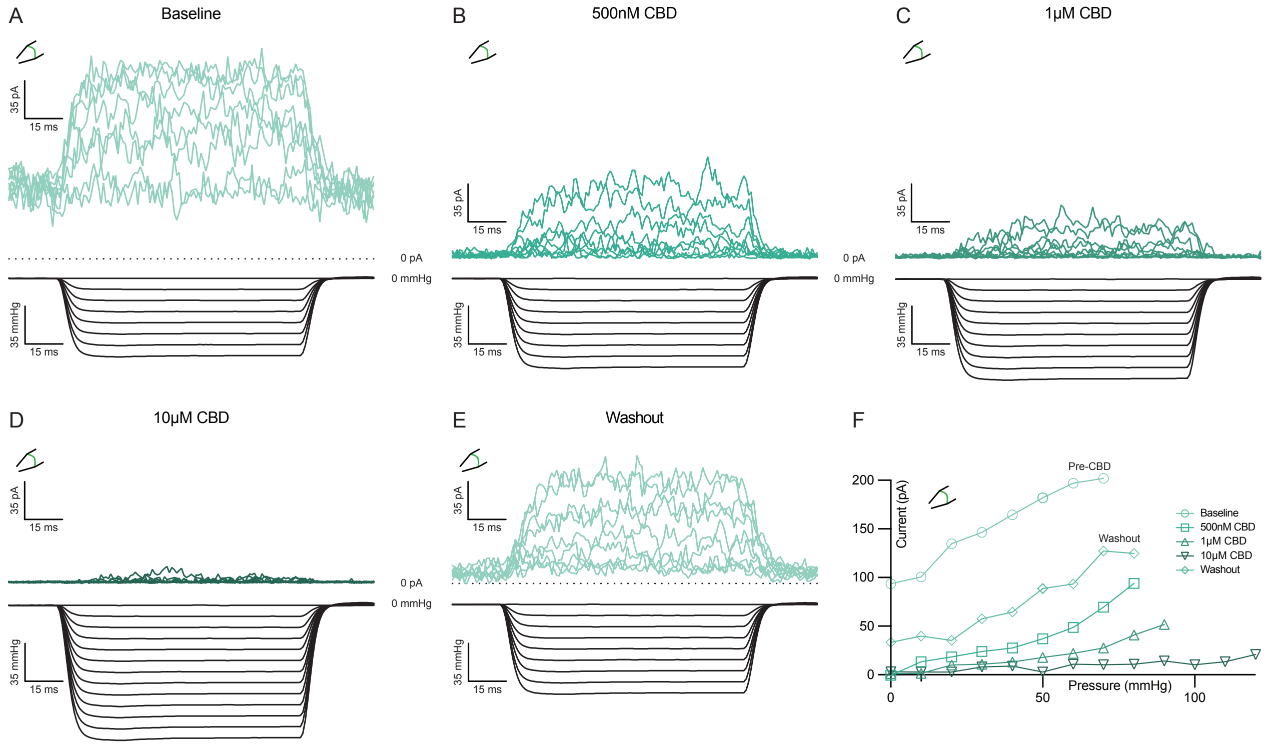

Supplementary Figure 2 – CBD inhibition reduces mechanical activation of TRAAK.
(a-e) Example current traces from a TRAAK-containing excised patch in response to negative pressure at varying concentrations of CBD ranging from (a) 0μM, (b) 500nM, (c) 1μM, (d) 10μM, and (e) following washout. (f) Peak current versus pressure from data in (a-e).

bioRxiv preprint doi: https://doi.org/10.1101/2024.12.09.627564; this version posted December 10, 2024. The copyright holder for this preprint (which was not certified by peer review) is the author/funder, who has granted bioRxiv a license to display the preprint in perpetuity. It is made available under aCC-BY 4.0 International license.

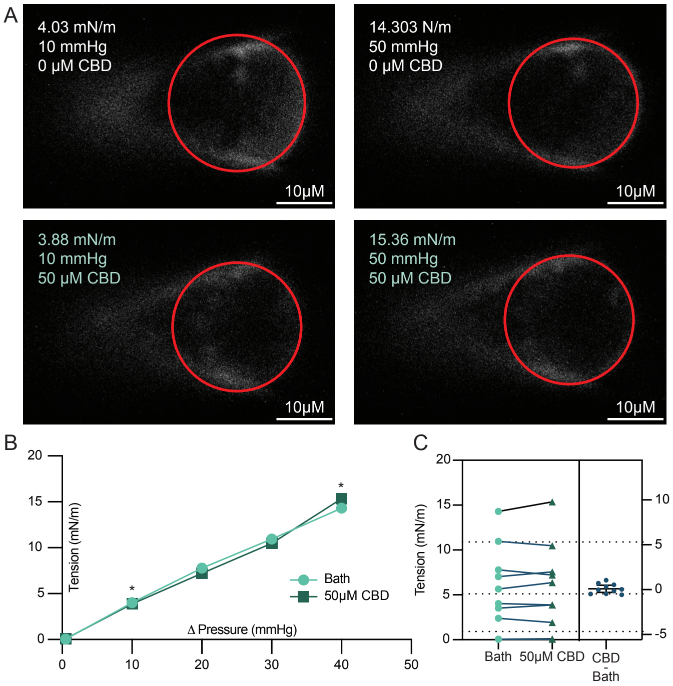

Supplementary Figure 3 – CBD treatment does not alter membrane tension.
(a) Representative images of membrane patches under -10 (left) and -50 mmHg (right) pressure in 0 µM CBD (top) and 50µM CBD (bottom). (b) Calculated membrane tension versus applied negative pressure in 0 µM CBD (light green) and 50 µM CBD (dark green). Asterisks indicate data from images shown in (a). (c) Comparison of tension generated at matching pressure steps. Data from 10 paired recordings from three different patches are shown ($p = 0.58$, two-tailed paired t-test, not significant).

bioRxiv preprint doi: https://doi.org/10.1101/2024.12.09.627564; this version posted December 10, 2024. The copyright holder for this preprint (which was not certified by peer review) is the author/funder, who has granted bioRxiv a license to display the preprint in perpetuity. It is made available under aCC-BY 4.0 International license.

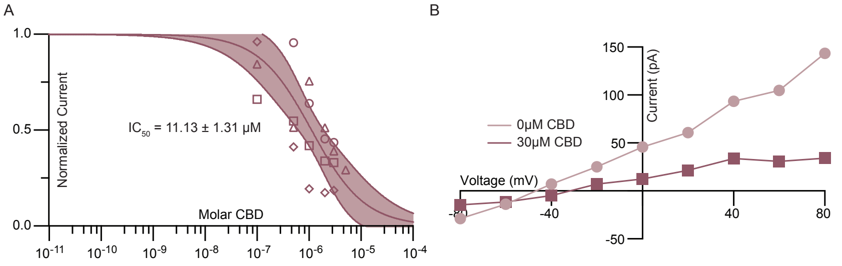

Supplementary Figure 4 – CBD inhibition of Danio rerio TREK-1.
(a) Dose-response curve. Data from individual patches are plotted with different shapes. Solid line is the fit to a four parameter logistic curve with 95% confidence interval shaded. IC_{50} = 11.1±1.3μM (mean±sem, n = 4 patches). (b) Representative current-voltage relationship from a Danio rerio TREK-1-containing patch before and after application of 30 μM CBD.

bioRxiv preprint doi: https://doi.org/10.1101/2024.12.09.627564; this version posted December 10, 2024. The copyright holder for this preprint (which was not certified by peer review) is the author/funder, who has granted bioRxiv a license to display the preprint in perpetuity. It is made available under aCC-BY 4.0 International license.

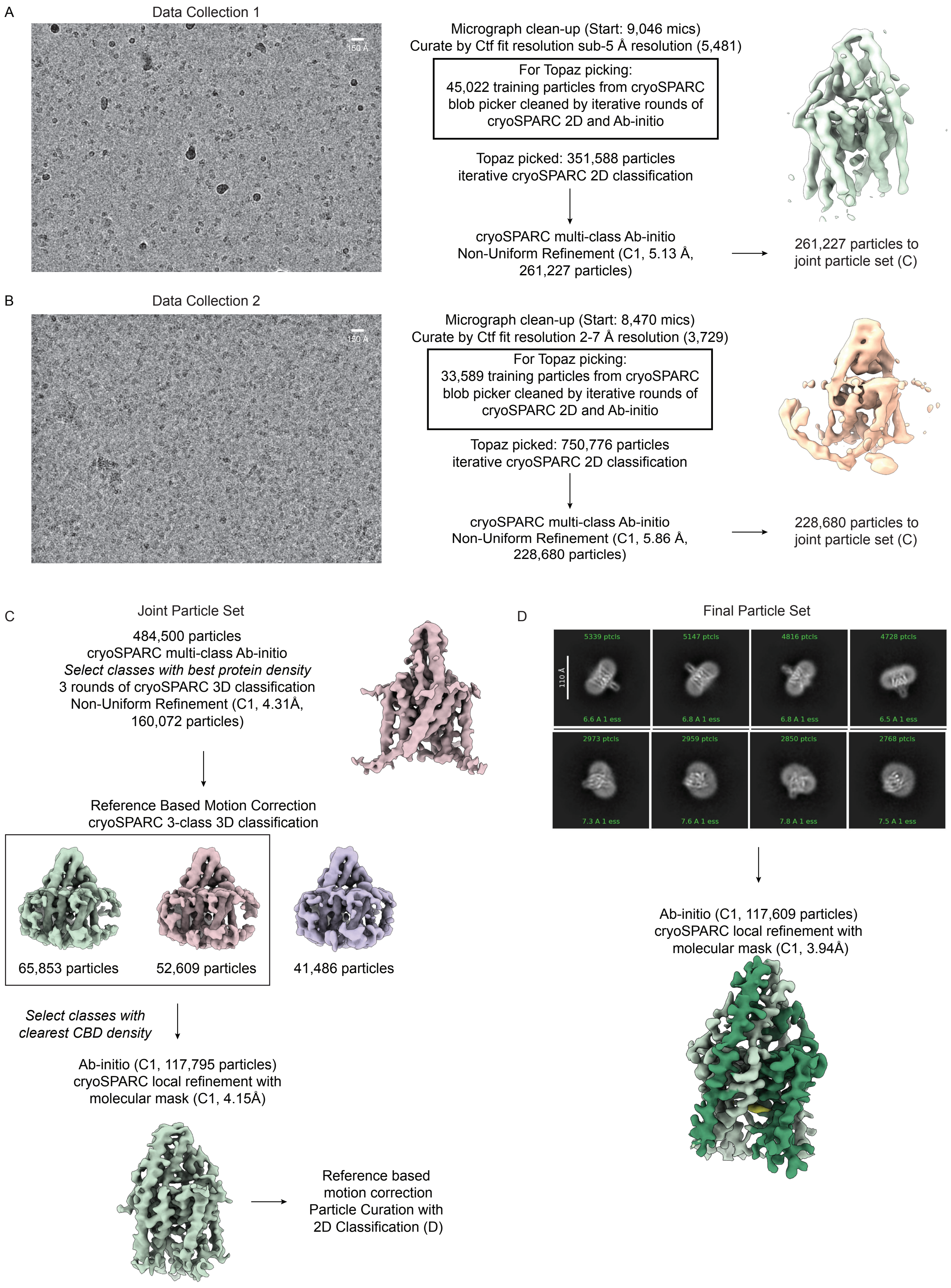

bioRxiv preprint doi: https://doi.org/10.1101/2024.12.09.627564; this version posted December 10, 2024. The copyright holder for this preprint (which was not certified by peer review) is the author/funder, who has granted bioRxiv a license to display the preprint in perpetuity. It is made available under aCC-BY 4.0 International license.

Supplementary Figure 5 – Cryo-EM data processing pipeline for TREK-1:CBD.

(a,b) Representative micrograph and initial cryo-EM data processing from each dataset, (c) cryo-EM data processing pipeline from merged particle stack, and (d) representative 2D class averages and reconstructed map from final particle stack.

bioRxiv preprint doi: https://doi.org/10.1101/2024.12.09.627564; this version posted December 10, 2024. The copyright holder for this preprint (which was not certified by peer review) is the author/funder, who has granted bioRxiv a license to display the preprint in perpetuity. It is made available under aCC-BY 4.0 International license.

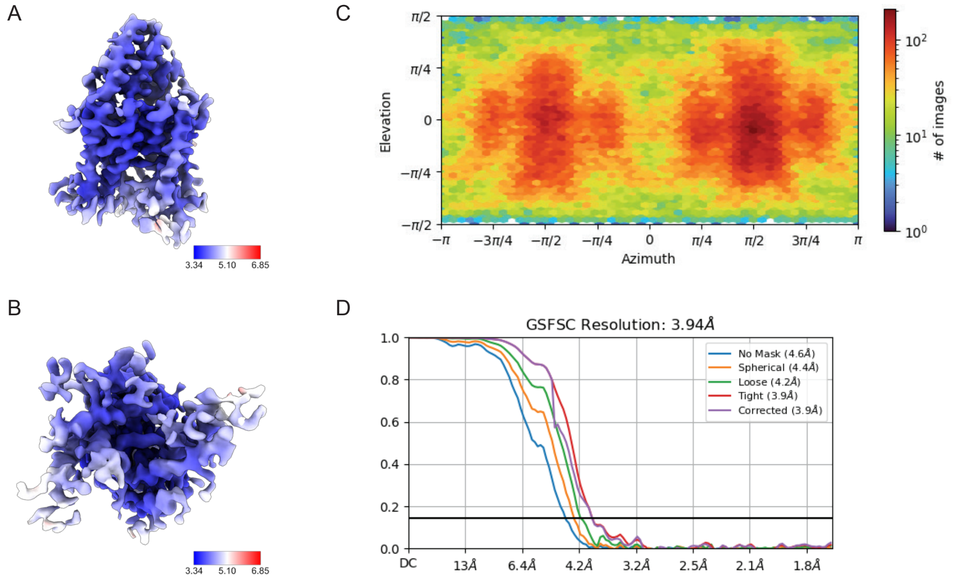

Supplementary Figure 6 – Cryo-EM structure validation for TREK-1:CBD
Final sharpened map colored by local resolution and viewed from (a) the membrane plane and (b) the cytoplasmic side. (c) View angle distribution of final particle stack. (d) Fourier shell correlation (FSC) versus resolution between half maps from final refinement.

bioRxiv preprint doi: https://doi.org/10.1101/2024.12.09.627564; this version posted December 10, 2024. The copyright holder for this preprint (which was not certified by peer review) is the author/funder, who has granted bioRxiv a license to display the preprint in perpetuity. It is made available under aCC-BY 4.0 International license.

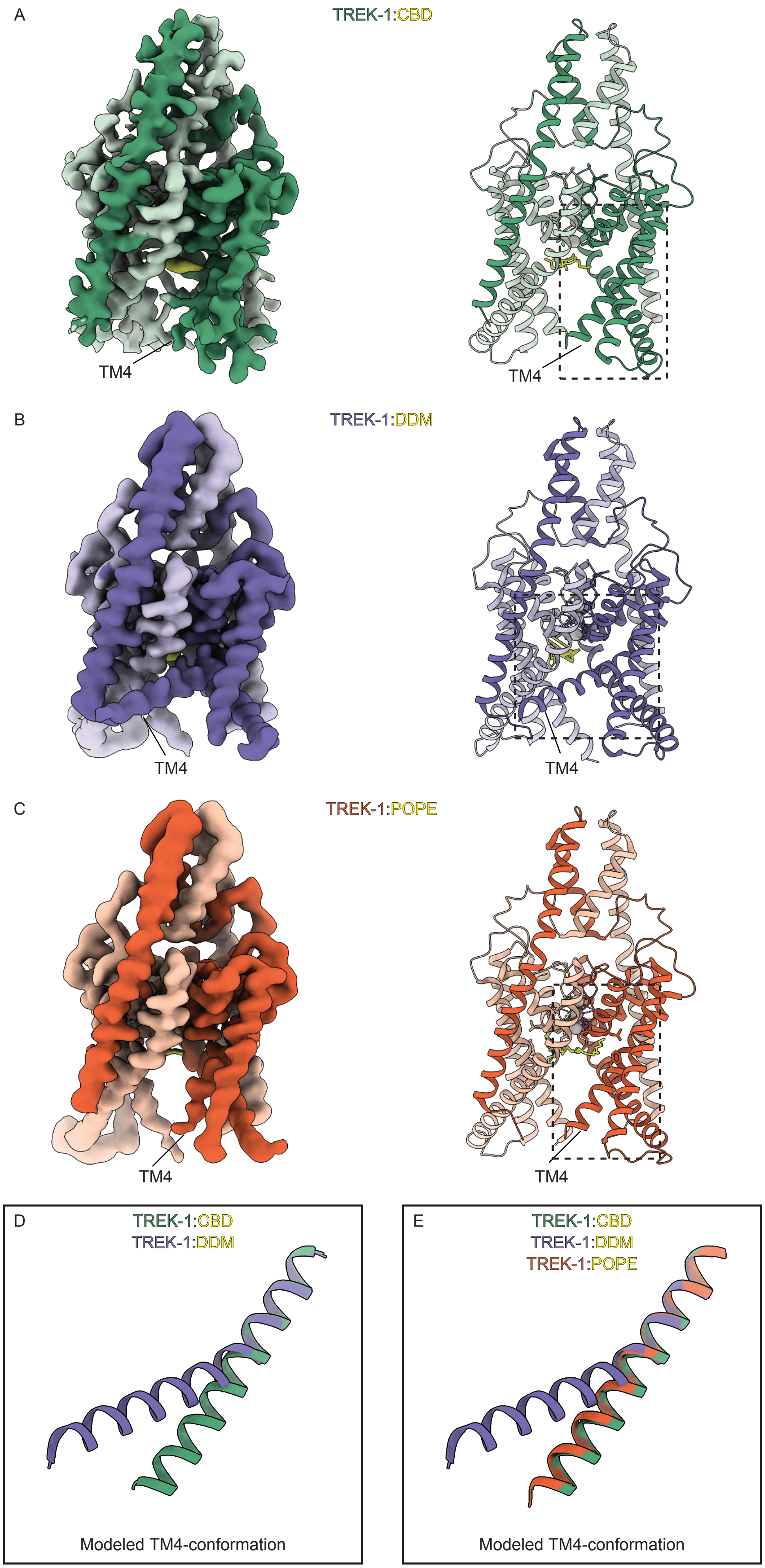

bioRxiv preprint doi: https://doi.org/10.1101/2024.12.09.627564; this version posted December 10, 2024. The copyright holder for this preprint (which was not certified by peer review) is the author/funder, who has granted bioRxiv a license to display the preprint in perpetuity. It is made available under aCC-BY 4.0 International license.

Supplementary Figure 7 – CBD-bound TREK-1 is stabilized in the TM4-down conformation.

(a) Final map and structure of TREK-1:CBD focused on TM4. (b) Final map and structure of apo TREK-1 focused on TM4. (c) Final map and structure of TREK-1:POPE focused on TM4. (d) comparison of TM4 conformation in apo TREK-1 (purple) and TREK-1:CBD (green) and (e) TREK-1:POPE.

bioRxiv preprint doi: https://doi.org/10.1101/2024.12.09.627564; this version posted December 10, 2024. The copyright holder for this preprint (which was not certified by peer review) is the author/funder, who has granted bioRxiv a license to display the preprint in perpetuity. It is made available under aCC-BY 4.0 International license.

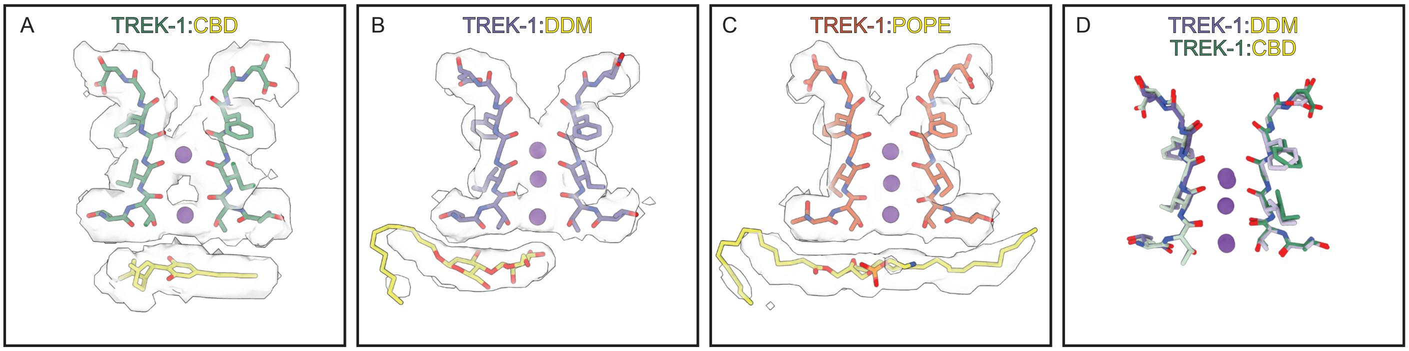

Supplementary Figure 8 – CBD-induced changes in the TREK-1 selectivity filter.
Comparison of the TREK-1 selectivity filter and K+ density from (a) TREK-1:CBD, (b) apo TREK:1, and (c) TREK-1:POPE structures. (d) Overlaid selectivity filters from apo TREK-1 (purple) and TREK-1:CBD (green) structures.
